# Selenium as a predictor of metabolic syndrome in middle age women

**DOI:** 10.18632/aging.204590

**Published:** 2023-03-21

**Authors:** Daria Schneider-Matyka, Anna Maria Cybulska, Małgorzata Szkup, Bogumiła Pilarczyk, Mariusz Panczyk, Agnieszka Tomza-Marciniak, Elżbieta Grochans

**Affiliations:** 1 Department of Nursing, Pomeranian Medical University in Szczecin, Szczecin 71-210, Poland; 2 Department of Animal Reproduction Biotechnology and Environmental Hygiene, West Pomeranian University of Technology, Szczecin 71-217, Poland; 3 Department of Education and Research in Health Sciences, Faculty of Health Sciences, Medical University of Warsaw, Warsaw 00-581, Poland

**Keywords:** selenium, metabolic syndrome, PPAR-γ, middle aged women

## Abstract

Background: Selenium plays an important role in metabolic homeostasis. It has been suggested that it may also affect the expression and activity of PPAR-γ. The aim of study was to analyze the relationships between these variables in the context of the health of women, for whom the risk of MetS increases with age.

Material and Methods: The study involved 390 women in middle age. The stages of study: a survey-based part; anthropometric measurements; analysis of biological material (blood) in terms of glycemia, triglyceride, HDL, and selenium levels, as well as genetic analysis of the PPAR-γ polymorphisms.

Results: It was found that selenium may moderate the effect of the G allele of the PPAR-γ gene on the occurrence of elevated waist circumference (OR=1.030, 95%CI 1.005-1.057, p=0.020); and the effect of the C (OR=1.077, 95%CI 1.009-1.149, p=0.026) and the G alleles (OR=1.052, 95%CI 1.025-1.080, p<0.000) on the odds of elevated blood pressure. Women in whom HDL levels were not significantly reduced, had higher selenium levels (p=0.007).

Conclusions: 1. The effect of selenium on MetS and its components has not been demonstrated. 2. The effect of individual alleles of the PPAR-γ gene on MetS and its components was not demonstrated. 3. The concentration of selenium may affect waist circumference in carriers of the G allele, and arterial hypertension in carriers of the C and G alleles by affecting the expression of PPAR-γ. 4. Higher selenium concentrations increased the odds of higher HDL levels in the group of subjects meeting the MetS criteria.

## INTRODUCTION

Metabolic syndrome (MetS) is a widespread clinical entity that has become almost a global epidemic [1]. MetS increases the incidence of type 2 diabetes (T2D) and cardiovascular disease (CVD), which poses a great threat to public health and the entire social economy [2]. MetS often affects populations characterized by excessive food intake and lack of physical activity [3]. Metabolic and genetic susceptibility is also a potential MetS risk factor [4, 5]. Abdominal obesity, elevated serum triglyceride and glucose levels, hypertension and an unstable ratio of low- and high-density lipoprotein concentrations are components of MetS [6]. Previous hypotheses suggested that MetS is initiated by insulin resistance (IR) [7]. IR causes hyperglycemia, but it is still not confirmed whether it also affects other metabolic factors [8]. People with marked upper body obesity are more vulnerable to MetS [9]. It has also been suggested that obesity may be a major cause of MetS due to the close relationship between obesity and all components of MetS [10, 11].

Excess energy intake and comorbid obesity are believed to be major risk factors for this syndrome. Lifestyle changes have been shown to reverse metabolic risk factors [12].

The aging period in a woman’s life has been identified as a high-risk stage for weight gain. Weight gain associated with approaching menopause is a consequence of low estrogen levels due to the progressive loss of ovarian function. Moreover, changes in the hormonal environment, chronological aging, and a decrease in physical activity, combined with a Western dietary pattern, can contribute to an increase in total body fat and waist circumference. A larger waist circumference is an independent risk factor for cardiovascular and metabolic diseases [13].

In recent years, there has been an increasing interest in essential trace elements and minerals [14], although these make up a low percentage of total body weight (less than 0.01%) [15]. There are 20 trace elements essential for maintaining human physiological homeostasis [16]. They include selenium (Se), which plays an important role in metabolic homeostasis [17–19]. The WHO has confirmed the role of selenium as an essential trace element [20]. It plays an important role in cardiovascular disease and cholesterol modulation [21–24]. Therefore, it can be assumed that selenium concentrations may be correlated with the occurrence of MetS. Selenium also plays a role in many biological processes, including inflammation [25], oxidative stress [26], and lipid metabolism [27]. It is incorporated into selenoproteins that have a broad spectrum of pleiotropic effects, ranging from immune-enhancing, antioxidant, and anti-inflammatory effects to the production of the active thyroid hormone [28]. Clinical data suggest that patients suffering from chronic liver diseases, such as steatosis and cirrhosis have significantly lower plasma selenium concentrations [29]. Selenium supplementation has been found to restore liver function [30]. It is mostly absorbed from food [31–33]. Both deficiency and excess of trace elements may adversely affect systemic homeostasis. The recommended daily intake of selenium is 50-200 μg/d [34]. It is only absorbed through the duodenum, which is related to frequently observed low levels of this element [35]. In addition, selenium intake varies widely around the world, ranging from 7 to 4990 μg per day, with an average of 60 μg per day in China and 40 μg per day in Europe [28]. Concentrations range from deficiency to toxic levels that cause a variety of symptoms, such as garlic breath, hair and nail loss, nervous system disorders, and poor dental health [36].

Peroxisome proliferator-activated receptors (PPARs) are transcription factors that belong to the nuclear hormone receptor family. Their main role is to regulate metabolism—normalize insulin resistance and fatty acid metabolism, and maintain glucose homeostasis. Through metabolism, they can also take part in the regulation of cell survival and proliferation, pathological states, and the development of metabolic diseases (e.g. diabetes, cancer). PPARs include three nuclear receptor isoforms, i.e. PPAR-α, PPAR-ϐ/δ, and PPAR-γ. PPAR-γ is an important mediator in energy balance and cell differentiation [37]. Research findings on PPAR-γ suggest that it is essential for lipid metabolism, adipogenesis, and maintenance of serum glucose homeostasis. It also affects the level of insulin resistance as well as inflammatory and tumorigenic processes [38, 39]. PPAR-γ may play a role in the development of hypertension by participating in the regulation of vascular tone [40]. Activation of PPAR-γ by rosiglitazone increases glucose uptake in muscle cells and adipocytes, and consequently lowers plasma glucose levels. This is associated with increased expression and translocation of glucose transporter 1 (GLUT1) and glucose transporter 4 (GLUT4) [41]. Lower triglyceride (TG) and higher high-density lipoprotein (HDL) levels may also potentiate the effect of PPAR-γ agonists on the development of MetS, atherosclerosis, and cardiovascular complications [42].

A clinical trial demonstrated the effect of selenium supplementation on the increased expression of PPAR-γ genes in lymphocytes of women with polycystic ovary syndrome (PCOS) [43]. Another animal study to investigate the role of the PPAR-γ/PI3K/Akt pathway in cadmium (Cd)-induced apoptosis showed that selenium antagonizes cadmium in the chicken pancreas [44]. On the other hand, selenium-enriched probiotics (Lactobacillus acidophilus and Saccharomyces cerevisiae) inhibit the expression of PPAR-γ genes, thereby improving lipid metabolism in mice fed with a high-fat diet [45]. The above data suggest that selenium may have a moderating effect on the expression and activity of PPAR-γ. Trace elements, including selenium, play an important role in metabolic homeostasis, so it is worth analyzing the relationships between these variables in the context of the health of women, for whom the risk of MetS increases with age.

The aim of this study was to search for a relationship between selenium concentrations and MetS, and to assess the impact of PPAR-γ on the incidence of MetS with regard to the moderating role of selenium.

## RESULTS

The study included a group of 390 women. Most of them (56.67%) had a higher education, 78.72% lived in a city with a population of more than 100,000, 73.33% were married. The vast majority (81.79%) of the women were non-smokers. 79.23% did not take any supplements, and 20.77% took supplements but not including selenium (Table 1).

**Table 1:** Characteristics of the study sample (N = 390).

	**n**	**%**
Education		
primary and vocational	27	6.92
secondary	142	36.41
tertiary	221	56.67
Place of residents		
rural area	36	9.23
small city (≤ 100 k)	47	12.05
big city (> 100 k)	307	78.72
Marital status		
married	286	73.33
informal relationship	41	10.51
single	63	16.15
Employment status		
unemployed	36	9.23
employed	354	90.77
Smoking		
no	319	81.79
yes	71	18.21
Supplements		
no	309	79.23
yes	81	20.77

The study sample was characterized in terms of MetS criteria. In our analysis, 71.28% of the participants had waist circumference ≥ 80 cm, 8.21% had blood glucose levels ≥ 100 mg/dl, 15.13% had triglyceride levels ≥ 150 mg/dl, 16.67% had HDL levels ≤ 50 mg/dl, 39.23% had diastolic blood pressure ≥ 85 mmHg and/or systolic blood pressure ≥ 130 mmHg. Less than one-fifth (18.46%) of the respondents met the criteria for MetS (Table 2).

**Table 2:** Characteristics of the study sample in terms of MetS components.

**Symptom**	**The 2009 IDF criteria for MetS diagnosis**	**n**	**%**
Waist circumference	[≥ 80 cm]	278	71.28
Fasting glycemia	[≥ 100 mg/dl or pharmacotherapy for diabetes]	32	8.21
Triglycerides	[≥ 150 mg/dl or related pharmacotherapy]	59	15.13
HDL cholesterol	[≤ 50 mg/dl or related pharmacotherapy]	65	16.67
Blood pressure	[≥ 85 mmHg and/or ≥ 130 mmHg or pharmacotherapy]	153	39.23
**MetS diagnosis (minimum 3 out of 5 components)**		72	18.46

Table 3 shows descriptive statistics concerning age, selected anthropometric variables, serum selenium concentration and individual MetS components. The mean age of the women was 52.59 ± 5.05 years. The mean values of anthropometric parameters were as follows: weight (72.22 ± 13.63 kg), height (164.79 ± 5.94), BMI (26.56 ± 4.59 kg/m^2^), fat mass (24.37 ± 8.52 kg), fat percentage (32.87 ± 5.74%), and visceral fat (7.04 ± 2.40). The mean selenium level was 99.43±19.74 μg/L. The mean values of MetS diagnostic criteria were: waist circumference (87.27 ± 12.24 cm), glycemic levels (86.09 ± 13.05 mg/dl), HDL (66.91 ± 16.40 mg/dl), triglycerides (107.56 ± 55.73 mg/dl), systolic blood pressure (119.24 ± 15.10 mmHg), and diastolic blood pressure (77.75 ± 9.69 mmHg) (Table 3).

**Table 3:** Characteristics of the study sample.

**Parameter**	**M**	**SD**	**Min**	**Max**	**CV [%]**
Age [years]	52.59	5.05	43.00	67.00	9.60
Weight [kg]	72.22	13.63	42.50	128.40	18.88
Height [cm]	164.79	5.94	147.00	180.00	3.60
Body Mass Index [kg/m^2^]	26.56	4.59	17.50	42.90	17.29
Body Fat Mass [kg]	24.37	8.52	9.20	63.20	34.97
Percent of Body Fat [%]	32.87	5.74	16.50	49.20	17.46
Visceral adipose tissue	7.04	2.40	3.00	15.00	34.07
Se^2-^ [μg/L]	99.43	19.74	53.16	182.00	19.85
**Components of MetS**					
Waist circumference [cm]	87.27	12.24	57.00	130.00	14.03
Fasting glucose [mg/dl]	86.09	13.05	62.80	194.40	15.16
HDL [mg/dl]	66.91	16.40	28.50	134.40	24.51
Triglycerides [mg/dl]	107.56	55.73	35.10	378.40	51.82
Systolic blood pressure [mmHg]	119.24	15.10	90.00	170.00	12.66
Diastolic blood pressure [mmHg]	77.73	9.69	50.00	105.00	12.47

M, mean; SD, standard deviation; CV, coefficient of variation; Min, minimum value; Max, maximum value.

Our analysis of the effect of serum selenium levels on particular components of MetS showed no association between these variables (p > 0.05) (Table 4).

**Table 4:** Comparative analysis of the mean selenium levels [μg/L g/L] in the groups singled out according to particular criteria for the diagnosis of MetS.

	**Se [μg/L g/L]**		**t_df=388_**	*p*-value*	d** (95%CI)
	**M**	**SD**			
Waist circumference [≥ 80 cm] n = 278	98.87	20.13	-0.879	0.380	0.01 (-0.12, 0.32)
Waist circumference [< 80 cm] n = 112	100.82	18.75			
Fasting glycemia [≥ 100 mg/dl or pharmacotherapy for diabetes] n = 32	101.00	20.69	0.467	0.641	-0.09 (-0.45, 0.28)
Fasting glycemia [< 100 mg/dl] n = 358	99.29	19.68			
Triglycerides [≥ 150 mg/dl or related pharmacotherapy] n = 59	102.11	21.12	1.129	0.259	-0.16 (-0.44, 0.12)
Triglycerides [< 150 mg/dl] n = 331	98.96	19.48			
HDL cholesterol [≤ 50 mg/dl or related pharmacotherapy] n = 65	95.64	17.63	-1.701	0.090	0.23 (-0.04, 0.50)
HDL cholesterol [> 50 mg/dl] n = 325	100.19	20.07			
Blood pressure [≥ 85 mmHg and/or ≥ 130 mmHg or pharmacotherapy] n = 153	100.60	21.38	0.941	0.348	-0.10 (-0.30, 0.11)
Blood pressure [< 85 mmHg and/or < 130 mmHg] n = 237	98.68	18.61			

M, mean; SD, standard deviation.

*Student’s two-tailed t-test, **Cohen’s *d* coefficient.

The effect of the PPAR-γ gene alleles on individual MetS diagnostic criteria was then analyzed. The analysis showed no association between the PPAR-γ gene alleles and individual MetS components (p > 0.05) (Tables 5A, 5B).

**Table 5A:** Analysis of the effect of the PPAR-γ gene C allele on MetS components.

**Diagnostic criteria for MetS**		**C(-)**		**C(+)**		*p*-value*
		**n**	**%**	**n**	**%**	
Waist circumference	< 80 cm	1	7.69	111	29.44	0.120
	≥ 80 cm	12	92.31	266	70.56	
Fasting glycemia	< 100 mg/dl	13	100.0	345	91.51	0.612
	≥ 100 mg/dl or pharmacotherapy for diabetes	0	0.00	32	8.49	
Triglycerides	< 150 mg/dl	11	84.62	320	84.88	1.000
	≥ 150 mg/dl or related pharmacotherapy	2	15.38	57	15.12	
HDL cholesterol	> 50 mg/dl	11	84.62	314	83.29	1.000
	≤ 50 mg/dl or related pharmacotherapy	2	15.38	63	16.71	
Blood pressure	< 85 mmHg and/or < 130 mmHg	6	46.15	231	61.27	0.387
	≥ 85 mmHg and/or ≥ 130 mmHg or pharmacotherapy	7	53.85	146	38.73	

*Fisher’s two tailed exact test.

**Table 5B:** Analysis of the effect of the PPAR-γ gene G allele on MetS components.

**Diagnostic criteria for MetS**		**G(-)**		**G(+)**		*p*-value*
		**n**	**%**	**n**	**%**	
Waist circumference	< 80 cm	80	30.89	32	24.43	0.194
	≥ 80 cm	179	69.11	99	75.57	
Fasting glycemia	< 100 mg/dl	237	91.51	121	92.37	0.847
	≥ 100 mg/dl or pharmacotherapy for diabetes	22	8.49	10	7.63	
Triglycerides	< 150 mg/dl	219	84.56	112	85.50	0.882
	≥ 150 mg/dl or related pharmacotherapy	40	15.44	19	14.50	
HDL cholesterol	> 50 mg/dl	220	84.94	105	80.15	0.251
	≤ 50 mg/dl or related pharmacotherapy	39	15.06	26	19.85	
Blood pressure	< 85 mmHg and/or < 130 mmHg	156	60.23	81	61.83	0.826
	≥ 85 mmHg and/or ≥ 130 mmHg or pharmacotherapy	103	39.77	50	38.17	

*Fisher’s two tailed exact test.

In further analysis, a logistic regression model was used to assess the odds of MetS and its components depending on the activity of PPAR-γ with regard to a moderating role of selenium.

In the case of waist circumference, the moderating effect of selenium levels was demonstrated in carriers of the G allele of the PPAR-γ gene (OR = 0.062; 95%CI 0050-819; p = 0.035). In women with high selenium levels, the presence of the G allele of the PPAR-γ gene significantly increased the odds of elevated waist circumference, while in those with low selenium levels, it did not increase the odds of elevated waist circumference at all. Thus, it can be assumed that the concentration of selenium moderates the effect of the G allele of the PPAR-γ gene on the occurrence of elevated waist circumference (OR = 1.030; 95%CI 1.005-1.057; *p* = 0.020).

At low selenium concentrations, the presence of the C allele of the PPAR-γ gene reduced the odds of elevated blood pressure, while at high selenium concentrations, this allele did not have such an effect on this parameter. It can be speculated that the concentration of selenium moderates the effect of the C allele on the odds of elevated blood pressure (OR = 1.077; 95%CI 1.009-1.149; *p* = 0.026).

Additionally, at low selenium concentrations, the presence of the G allele of the PPAR-γ gene decreased the odds of elevated blood pressure, while at high selenium concentrations, this allele increased the odds of elevated blood pressure. It can be speculated that the concentration of selenium moderates the effect of the G allele on the odds of elevated blood pressure (OR = 1.052; 95%CI 1.025-1.080; p < 0.000).

There were no relationships between the other MetS components and the activity of PPAR-γ moderated by selenium (Table 6).

**Table 6:** Influence of the activity of PPAR-γ moderated by selenium on the occurrence of MetS and its components (n = 390).

	**b**	**OR**	**-95%CI**	**+95%CI**	***p*-value**
Waist circumference					
Intercept	8.996				0.185
Allel C (+)	-6.677	0.001	0.000	693.823	0.322
Allel G(+)	-2.779	0.062	0.005	0.819	0.035
Se^2-^ [mg/L]	-0.067	0.936	0.828	1.058	0.287
Allel C (+)*Se^2-^ [μg/L]	0.051	1.053	0.932	1.189	0.409
Allel G (+)*Se^2-^ [μg/L]	0.030	1.030	1.005	1.057	0.020
Fasting glycemia					
Intercept	-15.744				0.000
Allel C (+)					
Allel G(+)	-1.942	0.143	0.003	6.505	0.318
Se^2-^ [mg/L]	-0.004	0.996	0.973	1.020	0.768
Allel C (+)*Se^2-^ [μg/L]					
Allel G (+)*Se^2-^ [μg/L]	0.019	1.019	0.983	1.056	0.310
Triglycerides					
Intercept	-4.899				0.363
Allel C (+)	2.970	19.484	0.001	638971	0.576
Allel G(+)	-1.407	0.245	0.012	5.085	0.363
Se^2-^ [mg/L]	0.033	1.033	0.937	1.139	0.512
Allel C (+)*Se^2-^ [μg/L]	-0.030	0.970	0.882	1.068	0.536
Allel G (+)*Se^2-^ [μg/L]	0.012	1.013	0.984	1.042	0.392
HDL					
Intercept	2.459				0.574
Allel C (+)	-2.618	0.073	0.000	313.079	0.540
Allel G(+)	-0.404	0.668	0.036	12.518	0.787
Se^2-^ [mg/L]	-0.050	0.952	0.865	1.047	0.310
Allel C (+)*Se^2-^ [μg/L]	0.033	1.034	0.941	1.135	0.487
Allel G (+)*Se^2-^ [μg/L]	0.008	1.008	0.979	1.039	0.573
Blood pressure					
Intercept	8.739				0.010
Allel C (+)	-8.177	0.000	0.000	0.192	0.014
Allel G(+)	-5.355	0.005	0.000	0.071	<0.001
Se^2-^ [mg/L]	-0.084	0.919	0.860	0.983	0.013
Allel C (+)*Se^2-^ [μg/L]	0.074	1.077	1.009	1.149	0.026
Allel G (+)*Se^2-^ [μg/L]	0.051	1.052	1.025	1.080	0.000
MetS diagnosis (minimum 3 out of 5 components)					
Intercept	3.324				0.428
Allel C (+)	-4.566	0.010	0.000	32.480	0.266
Allel G(+)	-2.291	0.101	0.006	1.722	0.113
Se^2-^ [μg/L]	-0.051	0.951	0.870	1.039	0.263
Allel C (+)*Se^2-^ [μg/L]	0.048	1.050	0.962	1.145	0.276
Allel G (+)*Se^2-^ [μg/L]	0.021	1.021	0.994	1.049	0.120

b, unstandardized regression coefficient; OR, odds ratio; CI, confidence interval.

Our analysis shows that the odds of reduced HDL levels in MetS subjects decrease as serum selenium concentrations increase (OR = 0.962; 95%CI 0.935-0.990) (p = 0.007) (Table 7).

**Table 7:** Relationship between selenium levels [μg/L] and the odds of developing MetS components (group with MetS; n = 72).

	**b**	**OR**	**-95%CI**	**+95%CI**	***p*-value**
Waist circumference					
Intercept	7.848	2560.962	0.674	75.950	0.062
Se^2-^ [μg/L]	-0.033	0.968	0.906	1.034	0.334
Fasting glycemia					
Intercept	-1.223	0.294	0.026	3.334	0.323
Se^2-^ [μg/L]	0.005	1.005	0.981	1.028	0.699
Triglycerides					
Intercept	-0.783	0.457	0.042	4.933	0.519
Se^2-^ [μg/L]	0.011	1.011	0.987	1.034	0.374
HDL					
Intercept	4.406	81.968	4.310	1558.820	0.003
Se^2-^ [μg/L]	-0.039	0.962	0.935	0.990	0.007
Blood pressure					
Intercept	0.202	1.224	0.052	28.985	0.901
Se^2-^ [μg/L]	0.013	1.013	0.982	1.046	0.416

b, unstandardized regression coefficient; OR, odds ratio; CI, confidence interval.

## DISCUSSION

MetS, which is a pathological state of energy distribution and storage, is widely regarded as a risk factor for cardiovascular disease and diabetes [46]. It is a constellation of metabolic disorders: abdominal obesity, insulin resistance, hypertension, glucose intolerance and dyslipidemia. Many factors are now recognized as contributing to the development of the MetS, among them trace elements that affect the structure of proteins, enzymes and complex carbohydrates. An imbalance of trace elements, including selenium, is an independent risk factor for MetS [47].

However, our study did not show any relationship between MetS and its components and serum selenium concentration. It is noteworthy that healthy women constituted the vast majority of the study sample, and less than 1/5 (18.46%) of the subjects met the diagnostic criteria for MetS. In addition, the majority of the participants had normal values of individual MetS components―elevated values were only found for waist circumference (71.28%) and blood pressure (39.23%). The results reported by other authors are not consistent.

Based on their study conducted in a group of MetS patients, Zhou et al. suggested a relationship between the concentration of selenium and MetS. The level of selenium was associated with central obesity, hypertriglyceridemia, hyperglycemia, as well as high blood pressure and HDL cholesterol [48]. Similar results were obtained by Yuan et al. [49]. A study by Wei et al. showed that dietary selenium intake was moderately negatively associated with MetS [50], and Zuleta et al. reported that selenium intake correlated negatively with triglyceride levels [51]. Another study showed that increased dietary selenium intake was associated with an increased risk of type 2 diabetes [52]—a finding that was not confirmed in the study by Raymna et al. [53]. Mutakin et al. found that the relationship between selenium intake and metabolic risk factors concerned only obese men [54]. According to Rayman et al., selenium deficiency significantly increases the risk of cardiovascular disease by reducing the concentration and activity of selenoproteins, which act as predictors of cardiovascular events [28]. Mirdamadi et al. provided evidence that most patients with heart disease have much lower levels of selenium in the blood and heart than healthy people [55].

In our investigation, the effect of the PPAR-γ gene alleles on the components of MetS was analyzed, but no relationship between these variables was noted.

We also evaluated the odds of MetS and its individual components depending on the PPAR-γ activity with regard to selenium as a moderator. We found that high levels of selenium may moderate the activity of the G allele of the PPAR-γ gene, contributing to the occurrence of elevated waist circumference. At low selenium levels, the G allele did not increase the odds of higher waist circumference.

Dtfeld and Horst-Sikorsta believe that the presence of the G variant of the PPAR-γ gene is a protective factor against obesity, however they did not take into account the role of selenium [56]. Cole et al., on the other hand, concluded that carriers of the C/G genotype are predisposed to elevated BMI values and obesity [57]. Swarbrick et al. did not note any association between the C/G genotype and obesity, hypertension or diabetes, but found that lipid metabolism disorders were more likely to affect obese carriers of the G allele. However, the authors of the above studies did not take into account the role of selenium [58].

Furthermore, our research indicates that high selenium levels may moderate the activity of both the G allele and the C allele of the PPAR-γ gene, thus contributing to elevated blood pressure.

Our findings partially confirm those reported by Szkup et al. Although these authors did not take into consideration the role of selenium, they demonstrated that the C/C genotype of the PPAR-γ rs1801282 variant was associated with elevated blood pressure in women aged 45-60 years [59]. Contradictory results were obtained by Ostgren et al., who also did not consider the role of selenium. In their study, the PPAR-γ C/G mutation was shown to be associated with lower diastolic blood pressure in humans [60]. Other findings indicate that PPAR-γ is involved in metabolic processes, such as abdominal obesity, insulin sensitivity, and lipid metabolism [61–66].

According to Nido et al., selenium supply reduces the PPAR-γ expression, positively affecting lipid metabolism in mice fed a high-fat diet [45]. This, however, is not supported by our findings, which show that subjects (narrowed down in this part of the study to the group meeting MetS criteria (n = 72)) with higher selenium concentrations have the odds of elevated HDL.

Inconsistency of the study results presented above may be due to the complex form and use of different diagnostic criteria for MetS. Other potential causes of the conflicting results concerning the relationships between selenium, PPAR-γ, and MetS are study samples differentiated in terms of selenium concentration, age, sex, and pharmacological treatment.

The presented research has some limitations. The study sample included an overwhelming majority of healthy women. Although the research was conducted among middle aged women, for which the risk of adverse metabolic changes is higher, the subjects meeting the MetS criteria constituted just 1/5 of the entire group, which may have influenced the results of the study. For a broader analysis of the relationship between selenium, PPAR-γ activity and the pathogenesis of MetS, research should be continued in a larger group of women meeting the MetS criteria, with the inclusion of healthy controls.

Moreover, the study group was recruited in the local environment through advertisements in the local press as well as information leaflets and posters placed in public places. Despite our efforts, there is a risk that the respondents do not constitute a representative sample, which may have influenced the results of this study.

### Clinical implications

Recently, optimizing selenium intake in the population to prevent diseases associated with selenium deficiency or excess has been an important issue in modern health care worldwide. Our study suggests the influence of selenium levels on some components of MetS, such as waist circumference, blood pressure and HDL concentration. Thus, serum selenium concentration could be considered as one of the factors affecting some components of MetS.

## CONCLUSIONS

1. The effect of selenium on MetS and its components has not been demonstrated.
2. The effect of individual alleles of the PPAR-γ gene on MetS and its components was not demonstrated.
3. The concentration of selenium may affect waist circumference in carriers of the G allele, and arterial hypertension in carriers of the C and G alleles by affecting the expression of PPAR-γ.
4. Higher selenium concentrations increased the odds of higher HDL levels in the group of subjects meeting the MetS criteria.

## MATERIALS AND METHODS

### Organization and course of study

The study was conducted in northwestern Poland among 390 middle aged women. Recruitment was based on advertisements in the local press and information posters left in public places.

It consisted of three stages. The first part was survey-based and performed using a questionnaire concerning sociodemographic data, selected medical information on chronic diseases, pharmacotherapy for hypertension, hypertriglyceridemia, hyperglycemia, and excessively high HDL levels, as well as diseases related to cigarette and alcohol addiction. The criteria for inclusion in the study were female sex, no history of selenium supplementation, no inflammatory, psychiatric or cancer diseases, and no alcohol abuse. Consumption of less than 20g of pure alcohol per day or occasional consumption of no more than 40g of pure alcohol and a declaration of at least two days of abstinence from alcohol per week was taken as the norm [67]. Those who failed to meet these criteria were excluded.

### Anthropometric measurements

In the second part, anthropometric measurements were taken using a Tanita MC780 MA body composition analyzer. The waist was measured in a standing position between the lower edge of the ribcage and the upper edge of the iliac crest at the end of a gentle exhalation.

BMI was calculated according to the formula: weight in kilograms divided by height squared in meters (kg/m^2^) [68].

The normal percentage of body fat for women between the ages of 40-59 is 23% to 34%, while for women aged 60-79 it is 24-36%. The normal values for visceral adipose tissue range from 1-12 [69].

### Blood pressure measurement

Blood pressure was measured in a sitting position with a manual blood pressure cuff. The cuff was tightly wrapped around the patient’s right arm at heart level.

### Laboratory analysis

In the third part of the study, biological material (blood) was collected from a venous vessel according to the procedure for collecting, storing and transporting biological material from a peripheral vein. Blood was drawn between 7.00 a.m. and 9.30 a.m. after an overnight fast and a 10-minute rest in a sitting position. The biological material was collected in two Vacutainer tubes (Sarstedt, Nümbrecht, Germany): the first with 1 g/L K2, ethane-1,2-diyl dinitrilotetraacetic acid, and the second for serum biochemical analysis (7ml). Fasting glycemia, triglyceride, HDL, and selenium levels were measured. DNA was then isolated for genetic analysis of the PPAR-γ rs1801283 polymorphism. Genomic DNA was extracted from whole blood following standard procedures. All genotyping was based on the real-time fluorescence resonance energy transfer performed using the Light Cycler System 1.0 (Roche Diagnostic, Poland). Gene polymorphisms were determined under the following conditions: polymerase chain reaction (PCR) was performed with 50 ng DNA in a total volume of 20 ml containing 2 ml reaction mix, 0.5 mM of each primer, 0.2 mM of each hybridization probe and 2 mM MgCl2 for 35 cycles of denaturation (95° C for 10min), annealing (60° C for 10 seconds), and extension (72° C for 15 seconds) as recommended by the manufacturer. After amplification, a melting curve was generated by holding the reaction at 40° C for 20 seconds, and then heating slowly to 85° C. The fluorescence signal was plotted against temperature to give a melting curve for each sample. The polymorphisms were determined by analysis of the melting curves. In the PPAR-γ rs1801282 (C>G) polymorphism, peaks were obtained at 53.14° C for the G allele and at 62.12° C for the C allele.

Biological material for DNA analysis was stored and transported in accordance with the procedures of the Quality Management System of the Genetic Laboratory, the Department of Psychiatry (according to the EN 15189 standard).

The acceptable range for selenium concentration was between 93-121 μg/L. Selenium content was determined by the spectrofluorimetric method using 2,3-diaminonaphthalene (the Shimadzu RF-5001 PC). The samples were subjected to wet digestion in a mixture of concentrated acids HNO_3_ (230° C, 180 min.) and HClO_4_ (310° C, 20 min.). The measurement was performed at the emission wavelength of 518 nm and the excitation wavelength of 378 nm.

### Metabolic syndrome―diagnostic criteria

Based on the classification proposed by the International Diabetes Federation (IDF), we accepted the following diagnostic criteria for MetS in women:

- waist circumference ≥ 80 cm;
- serum triglyceride levels ≥ 150 mg/dL [1.7 mmol/L] or lipid-modifying pharmacotherapy;
- HDL-C levels ≤ 50 mg/dl [1.3 mmol/l] or lipid-modifying pharmacotherapy;
- systolic blood pressure ≥ 130mmHg and/or diastolic blood pressure ≥ 85 mmHg or pharmacological treatment of hypertension;
- fasting serum glucose ≥ 100 mg/dL [5.6 mmol/L] or diabetes pharmacological treatment.

MetS was diagnosed when three or more of the above criteria were met [70]. If the women took drugs to modify lipids or carbohydrate metabolism, they were regarded as having selected MetS components. Information was obtained from the questionnaire responses.

### Statistical analysis

The measurements for categorical and quantitative variables were presented using descriptive statistics. For quantitative variables, parameter of central tendency (mean (M)), measures of variation (standard deviation (SD) and coefficient of variation (CV)) and range (minimum-maximum) were calculated. Number (N) and frequency (%) were calculated for categorical variables.

Statistical inference was based on null hypothesis testing. Differences in the mean level of selenium depending on the diagnosis of individual MetS criteria were estimated using Student’s t-test. The effect size was determined using Cohen’s *d* coefficient.

Differences in the frequency of particular alleles of the PPAR-γ gene depending on the diagnosis of individual MetS criteria were estimated using Fisher’s two tailed exact test.

The impact of selenium on the odds of developing MetS components was estimated using a logistic regression model. The effect of the PPAR-γ gene alleles on the development of MetS components was also assessed taking into account the moderating role of selenium. The regression model parameters were estimated using the Rosenbrock method with asymptotic standard errors. All independent variables were entered into the regression model simultaneously. The odds ratio with 95% confidence interval was determined for each variable.

All calculations were performed with the Statistica™ 13.3 software (TIBCO^®^ Software, Palo Alto, CA, USA). For all analyses, the p-level under the 0.05 was considered statistically significant.
